# Radio antipodal number of honeycomb derived networks

**DOI:** 10.1038/s41598-022-23618-7

**Published:** 2022-11-08

**Authors:** S. Gomathi, P. Venugopal

**Affiliations:** 1 Department of Mathematics, Sri Sivasubramaniya Nadar College of Engineering, Kalavakkam, Tamil Nadu 603110 India; 2 Mathematics, School of Science and Humanities, Shiv Nadar University Chennai, Kalavakkam, Tamil Nadu 603110 India

**Keywords:** Engineering, Mathematics and computing

## Abstract

The communication in a wireless network mainly depends on the frequencies or channels assigned to them. The channels must be assigned to all the transmitters in the network without interference for effective communication. This problem is said to be a channel (frequency) assignment problem (CAP). With the limited availability of channels, CAP has become a challenging problem. This problem is modeled as a graph, where each transmitter is represented by a vertex, and two vertices are adjacent when their corresponding transmitters are close. The labelling technique in graph theory has played an important role in solving CAP, thereby the time and cost will be saved. In radio antipodal labeling, the channels were reused again for the antipodal vertices. It will reduce the usage of the number of channels, with minimum interference. Hence it is a better labeling compared to other labelings. It is a mapping $\tau$ from the vertex set of a graph *T* to the set of natural numbers such that the condition $d(\alpha ,\eta )+\mid \tau (\alpha )-\tau (\eta )\mid \ge diam(T)$, is satisfied. The span of the antipodal labeling $\tau$ is the maximum label allotted in a graph and is given by $sp(\tau )=max\{\mid \tau (\alpha )-\tau (\eta )\mid :\alpha ,\eta \in V(T)\}$. The lowest value of all the spans of the antipodal labeling of graph *T* is said to be radio antipodal number. It is denoted by *an*(*T*). The value of the minimum span gives the bandwidth or spectrum of the channels. The honeycomb network plays an important role in communication engineering because of its structure. In this paper, the bounds of the antipodal number of honeycomb derived networks—triangular and rhombic honeycomb were obtained and represented graphically. These bounds give the optimum number of channels (bandwidth) needed for these honeycomb derived networks for effective communication without interference.

## Introduction

Wireless communication has a lot of applications like radio and TV broadcasting, satellite communication, mobile networks, and so on^1^. Effective communications in a network depend on the channel (frequency) allotted to them from the available bandwidth (frequency spectrum). Due to the widespread usage of wireless services for mobile networks, the number of channels gets saturated at a faster rate and couldn’t meet the growing demand for bandwidth^2,3^.

The difficulty of assigning channels (radio frequencies) to transmitters in a network at different locations without interference using the available bandwidth is known as the *channel (frequency) assignment problem* (CAP). This problem was first formulated by Hale^4^ as a graph colouring problem in 1980, who introduced the notion of T-Coloring. The problem is solved by the graph labeling technique. Consider a network consisting of *n* stations (or transmitters). The task is to assign to each station, a channel (non-negative integer) without interference. The interference is closely associated to the geographical position of the stations, the smaller the distance between two stations, the interference becomes stronger. To avoid stronger interference, the difference between the channels assigned to the stations must be large. This problem is modeled as a graph, where each station is represented by a vertex, and two vertices are adjacent when their corresponding stations are close. The objective is to find a valid labelling for all the vertices such that the span (range) of the channels used is minimized^5,6^.

The *labeling* of a graph *T*^7^ is nothing but assignment of natural numbers (labels) to all the nodes or edges or both, satisfying certain conditions. Chartrand et al.^8^ initiated the study of radio labeling of graphs in 2001 as they were encouraged by regulations for frequency (channel) assignments to transmitters of FM radio stations. A *radio labeling* is a mapping $\tau$ from the nodes of a graph *T* to some subset of non-negative integers such that $d(\alpha ,\eta )+\mid \tau (\alpha ) - \tau (\eta )\mid \ge diam(T)+1$, where *diam*(*T*) is the diameter of *T*. The maximum label assigned to graph nodes is said to be the *span* of *T* and is given by $sp(\tau )=max\{\mid \tau (\alpha )-\tau (\eta )\mid :\alpha ,\eta \in V(T)\}$.

Let $k (k \ge 1)$ be an integer. A *radio* *k*-*labeling* of a graph *T* is an assignment of positive integers to the nodes of *T* such that $d(\alpha ,\eta )+\mid \tau (\alpha ) - \tau (\eta )\mid \ge k+1$. Radio labeling is said to be a *k*-*labeling* when $k=diam(T)$. When $k=diam(T)-1$, it is said to be radio antipodal labeling. In other words, a *radio antipodal labeling* of a graph *T* is a mapping $\tau :V(T)\rightarrow \{1,2,\ldots \}$ such that $d(\alpha ,\eta )+\mid \tau (\alpha ) - \tau (\eta )\mid \ge diam(T)$. The *radio antipodal number* of *T*, *an*(*T*) is the minimum of all spans of radio antipodal labeling of *T*. In radio antipodal labeling, if $d(\alpha ,\eta )=diam(T)$, then $\alpha ,\eta$ may receive the same labeling.

Obtaining the radio number of generic graphs is computationally challenging and it is *NP*-hard to determine, the radio number of general graphs of diameter 2 and its complexity is unknown in general^9^. The radio and antipodal number for different types of graphs has been studied by several authors^10–14^. The radio number of Triangular and Rhombic Honeycomb Networks are determined in^15^. The antipodal number of cycles has been established by Juan and Liu^16^. The radio number and antipodal number for the hypercube were determined in^17^. The antipodal number of some powers of cycles was discovered by Saha^18^. The radio antipodal labeling of the full binary tree was investigated in^19^. Some more results in radio number and radio antipodal number can be referred to^20–26^.

**Figure 1:**
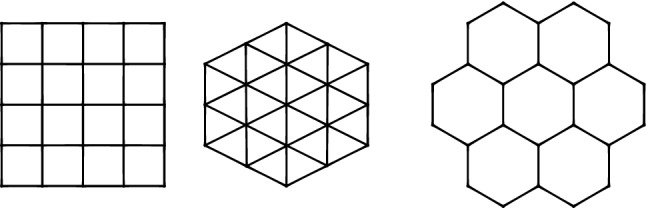
Regular plane tessellations.

Paul et al.^27^ mentioned three types of regular plane tessellations or patterns: triangular, square, and hexagonal as shown in Fig. 1. These tessellations are the basis for the design of any communication network. The honeycomb structure can be imagined as a multiprocessor interconnection network that was developed in a recursive manner utilising hexagonal tessellation. A honeycomb network is built by the arrangement of hexagons, in various ways^27,28^. The honeycomb mesh built in the form of a triangle is said to be a *triangular honeycomb* and the honeycomb mesh constructed in the form of the rhombus is said to be a *rhombic honeycomb*. The bounds of the radio antipodal number of the honeycomb-derived networks like triangular and rhombic honeycomb networks have been established in this article.

This paper is organized as follows. This paper consists of three sections. “Introduction”Section is introductory which gives a brief literature survey of radio labeling and radio antipodal labeling along with a few terminologies necessary for our study. In “The radio antipodalnumberfortriangularhoneycombnetwork”Section, the bounds of the antipodal number for triangular honeycomb network was determined. The "The radio antipodalnumberfortherhombichoneycombnetwork"Section gives the bounds of the antipodal number for rhombic honeycomb network followed by the Conclusion.

## The radio antipodal number for triangular honeycomb network

This section establishes the bounds of the antipodal number for triangular honeycomb network *THC*(*n*), $n>0$.

### Definition 2

^29^The *sum* of the distance between the vertex *r* to every other vertex *s* in *T* is denoted by *S*(*r*). Mathematically it is written as $S(r)=\sum _ {s\in T} d(r,s)$. The minimum value of all $S(r),\forall r\in T$ is said to be the *median* of *T*. It is denoted by *M*(*T*). That is $M(T)=min\{S(r):r\in T\}$. The *center* of *T* is the vertex *r*, for which *M*(*T*) is defined.

### Construction

^28^ *THC*(*n*) is created by keeping continuous hexagonal patterns within a triangle.

**Figure 2:**
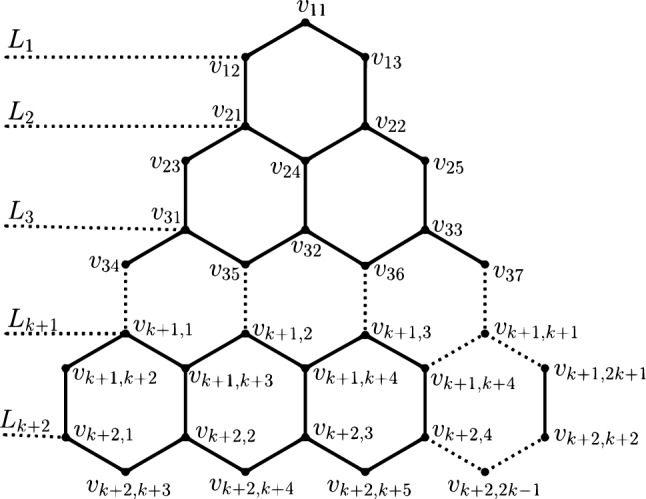
Different Levels of *THC*(*n*).

It is a downward connecting honeycomb network. Consider a hexagon. Add two hexagons at the base of the first hexagon so that both the hexagons have a common edge, as shown in Fig. 2. These two hexagons added are considered to be in layer one. Similarly, this network can be constructed up to $(n-1)$ layers, having $(n+1)$ hexagons in the $n^{th}$ layer, *n* hexagons in the $(n-1)^{th}$ layer, and so on.

The $n{th}$ dimension of triangular honeycomb network is represented by *THC*(*n*). It has $n^2+6n+6$ nodes and $\frac{3}{2}(n^2+5n+4)$ edges.

**Figure 3:**
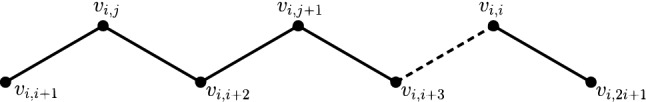
*i* *th* level of *THC*(*n*).

### Remark 2

^15^ *THC*(*n*) has $n+2$ levels. It can be divided into $L_{k}$ and $L_{r}$, for $1\le k\le n+1$ and $r=n+2$. The $k^{th}$ level vertex set is given by $v_{k,j}$ $1\le j\le 2k+1$, and the vertex set in $r^{th}$ level is $v_{r,j}$ $1\le j\le 2n+3$. The different levels of *THC*(*n*) is shown in Fig. 2. The nodes $v_{i,i+1},v_{i,j},v_{i,i+2},v_{i,j+1}\ldots v_{i,2i+1}$ in *THC*(*n*) are said to be in level *i*, as shown in Fig. 3.

In *THC*(*n*), $n>0$, $3(n+1)$ pairs of vertices are diametrically opposite.

### Lemma 2

The diameter of *THC*(*n*), $n > 0$, is $2n+3$.

### Proof

By the structure of the triangular honeycomb network, the nodes at the corner are at maximum distance from one another. To prove this Lemma, let us choose any two of these nodes. Without loss of generality, choose the node $v_{11}$ from the first level and the node $v_{n+2,2n+3}$ from the $(n+2)^{th}$ level.

Consider a node $v_{n,2n+3}$ in the path joining $v_{11}$ and $v_{n+2,2n+3}$. The shortest path between $v_{11}$ and $v_{n,2n+3}$ is given by

$v_{11} \rightarrow v_{13}\rightarrow \cdots \rightarrow v_{n-1,2n+3}\rightarrow v_{n,2n+3}$. There are 2*n* nodes in this path. Hence, $d(v_{11},v_{n,2n+3} )=2n-1$. The path between $v_{n,2n+3}$ and $v_{n+2,2n+3}$ is defined by $v_{n,2n+3}\rightarrow v_{n+1,n+1}\rightarrow v_{n+1,2n+3}\rightarrow v_{n+2,n+2} \rightarrow v_{n+2,2n+3}$.

Hence, $$d(v_{n,2n+3},v_{n+2,2n+3} )=4$$.

Therefore, $$d(v_{11} ,v_{n+2,2n+3} )=d(v_{11} ,v_{n,2n+3} )+d(v_{n,2n+3} ,v_{n+2,2n+3})=2n-1+4=2n+3$$. $$\square$$

### Theorem 2.1

$$an(THC(n))\ge M(THC(n))+2n$$, $$n\ge 2$$.

### Proof

Let $\tau$ be optimum antipodal labeling of *THC*(*n*). We can associate to $\tau$ an ordering of the nodes of *THC*(*n*), by increasing their labels. That is, if $v_{11},v_{21} \ldots v_{n+2,1}$ are the nodes of *T* taken in this order, then $\tau (v_{11})\le \tau (v_{21})\le\cdots \le \tau (v_{n+2,1})$. By using Definition 2, the center of *THC*(*n*) can be found. Suppose the node *v* is the center of *THC*(*n*). Label it as 1. Starting from this node *v*, the remaining nodes of *THC*(*n*) are labeled, by applying the antipodal condition $d(\alpha ,\eta )+\mid \tau (\alpha ) - \tau (\eta )\mid \ge diam(THC(n))$. Then the span of $\tau$ is $max\{\mid \tau (\alpha ) - \tau (\eta )\mid :\alpha ,\eta \in V(THC(n)),\alpha \ne \eta \}$. Applying the method given by Cada et al.^30^, the lower bound of the antipodal number of *THC*(*n*) is obtained as $an(THC(n))\ge M(THC(n))+2n,n\ge 2$, where $M(THC(n))=\frac{1}{2}(29n^2-27n+46)$ for all $n>0$. $\square$

### Theorem 2.2

$$an(THC(1))=24$$.

### Proof

For *THC*(1), $\mid V(THC(1)) \mid = 13$, $\mid E(THC(1)) \mid = 15$ and $diam(THC(1))=5$. From Theorem 2.1, $M(THC(n))=\frac{1}{2} (29n^2-27n+46)$. For $n=1$, $M(THC(1))=\frac{1}{2} (29-27+46)=24$.

Therefore $$an(THC(1))\ge 24$$.

The following condition must be satisfied for all antipodal labeling

1 $$\begin{aligned} d(\alpha ,\eta )+\mid \tau (\alpha ) - \tau (\eta )\mid \ge diam(THC(1))=5. \end{aligned}$$

To get the minimum labeling, start the labeling from the center of *THC*(1). For *THC*(1), the median of the graph occurs at node $v_{24}$. Therefore the center of the node is $v_{24}$ hence label it as 1. The remaining nodes of *THC*(1) are labeled based on the maximum distance from the already labelled nodes, satisfying antipodal condition (1). From $v_{24}$ choose a node that is at maximum distance. Without loss of generality, we take node $v_{11}$ and label it as 3, i.e., $\tau (v_{11} )=3$. From $v_{11}$, the node $v_{35}$ is at maximum distance, hence label it as 4, i.e., $\tau (v_{35} )=4$. Proceeding like this, the remaining nodes of *THC*(1) are labeled in the following sequence $v_{35}\rightarrow v_{25}\rightarrow v_{23}\rightarrow v_{32}\rightarrow v_{22}\rightarrow v_{33} \rightarrow v_{12}\rightarrow v_{13}\rightarrow v_{31}\rightarrow v_{21}\rightarrow v_{34}$.

In this sequence, the last node $v_{34}$ receives the maximum labelling 24, which is the span of *THC*(1).

Hence, $$an(THC(1))=24$$. $$\square$$

### Theorem 2.3

For *THC*(*n*), $$n \ge 2$$

$$\begin{aligned} an(THC(n)) \le \left\{ \begin{array}{lll} 2n^{3}+9n^{2}+5n+10 &{} \text{ for } &{} n=2 \\ 2n^{3}+9n^{2}+7n+5 &{} \text{ for } &{} n>2. \end{array}\right. \end{aligned}$$

### Proof

The node(vertex) set of *THC*(*n*) is defined as $\{v_{k,j};1\le k\le n+1 ,1\le j\le 2k+1$ and if $k=n+2, 1\le j\le 2k-1\}$. By Lemma 2, the $diam(THC(n))=2n+3$. From Remark 2, the maximum number of nodes of *THC*(*n*) at diametric distance are $3(n+1)$. Let $\tau$ be the optimum antipodal labeling of *THC*(*n*) such that it satisfies the condition

2 $$\begin{aligned} \mid \tau (\alpha )-\tau (\eta )\mid \ge 2n+3-d(\alpha ,\eta ). \end{aligned}$$

Now take $\tau (v_{1,1} )=1$, $\tau (v_{1,2} )=diam(THC(n))$, and $\tau (v_{1,3} )=\tau (v_{1,2} )+diam(THC(n))-2$. Without loss of generality, we consider only three pairs of diametric opposite nodes, say $(v_{1,1},v_{n+2,2n+3})$, $(v_{1,2},v_{n+2,n+2})$, $(v_{1,3},v_{n+2,1})$ so that they receive the same labeling by antipodal condition. The remaining nodes of *THC*(*n*) are labeled as follows.

*Case (i):* $$L_k=\{v_{kj},2\le k < n+2\}$$.

   *Sub case (a):* If $j=1$, then $\tau (v_{kj} )= \tau (v_{k-1,2k-1} )+2n+3-(2k-1)$.

      If $$j=k+1$$, then $$\tau (v_{kj} )= \tau (v_{k,k})+2n+3-(2k-1)$$.

   *Sub case (b):* If $2\le j\le 2k+1$, $j\ne k+1$ , then $\tau (v_{kj} )= \tau (v_{k,j-1} )+2n+1$.

*Case (ii):* $$L_k=\{v_{kj},k=n+2, 1\le j\le 2k-1\}$$.

   *Sub case (c):* If $j=2$ , then $\tau (v_{kj} )= \tau (v_{k-1,k} )+2$ .

      If $$3\le j\le k-1$$, then $$\tau (v_{kj} )= \tau (v_{k,j-1} )+2n+1$$.

   *Sub case (d):* If $j=k+2$, then $\tau (v_{kj} )=\tau (v_{2,1} )+2$.

   If $$k+2 < j\le 2k-1$$, then $$\tau (v_{kj} )= \tau (v_{k,j-1} )+2n+1$$.

Now, let us prove that the above cases satisfies the antipodal condition (2).

*Case (1):* Let $\alpha ,\eta \in THC(n)$ be in the same level and $d(\alpha ,\eta )=1$.

Let $\alpha =u_{k1}$,$\eta =v_{k,k+1}, v_{k,k+2}$ and $v_{k-1,k}$, Clearly $\tau (\eta )\ge diam(THC(n))$. $\mid \tau (\alpha )- \tau (\eta )\mid \ge 2n+3-1=2n+2$. This implies that $\tau (\alpha )=2n+2+\tau (\eta )> 2n+3$. From sub case(b) for $2\le j\le 2k+1$ , $j\ne k+1$, $\tau (\alpha )=2n+3+\tau (\eta )-1$ $=2n+2+\tau (\eta )> 2n+3$. The proof is similar for sub cases (c) and (d). This is true for every other node on the same level.

*Case (2):* Suppose the nodes are in different levels and $d(\alpha ,\eta )=1$.

For $2\le k\le n+2$, the nodes are $(\alpha =u_{k,k+1},\eta =v_{k+1,1} ),(\alpha =u_{k,k+2},\eta =v_{k+1,k} ) \ldots (\alpha =u_{k,2k+1},\eta =v_{k+1,k+1})$. From sub case(a), $\mid \tau (\alpha )- \tau (\eta )\mid =2n+3-2k+1-1\ge 2n+3$, for any *k*. Similarly, the antipodal condition for all other sub cases can be verified.

*Case (3):* Consider $$2\le d(\alpha ,\eta )\le 2n+3$$.

Suppose $d(\alpha ,\eta )=2n+3.$ Let $\alpha =v_{11},\eta =v_{n+2,2n+3}$. By Lemma (2), $d(v_{11},v_{n+2,2n+3} )=2n+3$. Therefore $\tau (\alpha )-\tau (\eta )=0$, as the diametrically opposite nodes receive the same label.

   *Sub case (i):* Suppose the nodes are in the different levels.

   Consider $2\le d(\alpha ,\eta )\le 2n+2$. Let $\alpha =v_{k,j},\eta =v_{k+1,j},1\le j\le 2k+1$. Then $\mid \tau (\alpha )-\tau (\eta )\mid =2n+3-d(\alpha ,\eta )$. This

   implies$$\tau (\alpha )=2n+3-d(\alpha ,\eta )+\tau (\eta )>2n+3$$.

   *Sub case (ii):* Suppose the nodes are in the same level.

   Let $$\alpha =v_{kj},\eta =v_{k,j+1},1\le j\le 2k+1$$. Then $$\tau (\alpha )=2n+3-d(\alpha ,\eta )+\tau (\eta )>2n+3$$.

Hence the antipodal condition is satisfied for every pair of nodes of *THC*(*n*).

In *THC*(*n*), the node $v_{n+2,n+1}$ receives the maximum label and $\tau (v_{n+2,n+1} )= 2n^3+9n^2+5n+10$ , for $n=2$, and

$\tau (v_{n+2,n+1} )= 2n^3+9n^2+7n+5$, for $n>2$, which is the span of *THC*(*n*). Hence the upper bound of the antipodal number is

$$\begin{aligned} an(THC(n)) \le \left\{ \begin{array}{lll} 2n^{3}+9n^{2}+5n+10 &{} \text{ for } &{} n=2 \\ 2n^{3}+9n^{2}+7n+5 &{} \text{ for } &{} n>2. \\ \end{array}\right. \end{aligned}$$

$$\square$$

## The radio antipodal number for the rhombic honeycomb network

This section establishes the bounds of the radio antipodal number for rhombic honeycomb network *RHC*(*n*), $n\ge 1$.

### Construction

^28^ *RHC*(*n*) is created by keeping continuous hexagonal patterns within a rhombus.

**Figure 4:**
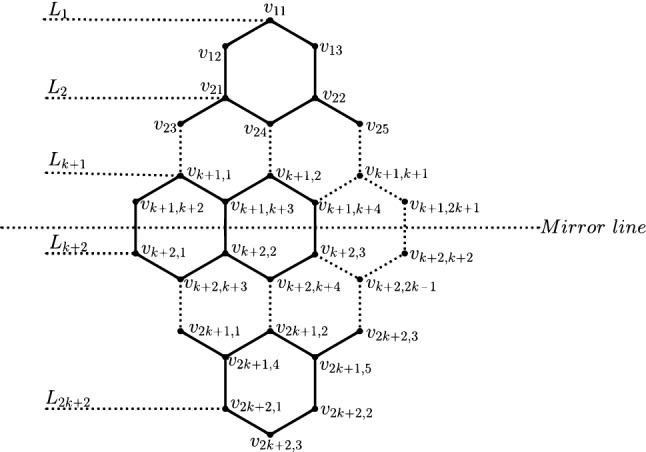
Different levels of *RHC*(*n*).

First let us construct (*RHC*(1)). Place 2 hexagons in series. keep one hexagon above and one hexagon below these two hexagons. This structure is said to be one dimensional rhombic honeycomb network, *RHC*(1). Similarly, the $n^{th}$ dimensional rhombic honeycomb network, *RHC*(*n*) can be constructed by having $(n+1)$ hexagons placed in series. Then $n,n-1, n-2,\ldots 2,1$ hexagon are added above and below these $(n+1)$ hexagons, taken in this order. See Fig. 4.

### Remark 3

^15^ The *level* (*L*) of *RHC*(*n*) are the nodes lying in every horizontal path taken from the left side of *RHC*(*n*) to the right side of *RHC*(*n*). There are $2(n+1)$ levels in *RHC*(*n*). These levels are classified into $L_k$ levels, for $1\le k\le n+1$ and $L_r$-levels, for $n+2 \le r \le 2n+2$, as shown in Fig. 4. The $L_r$-levels are nothing but the mirror image of $L_k$ levels. That is the levels from $(n+1)$ to $(2n+2)$ levels are symmetric to the first above $n+1$ levels. This implies, the number of nodes in the levels $L_{n+2},L_{n+3} \ldots L_{2n+2}$ is equal to the number of nodes in the levels $L_{n+1},L_n\ldots L_1$ taken in that order. The node(vertex) set of $i^{th}$ level is $\{v_{i,j}$, $1\le i\le n+1$,$1\le j\le 2i+1\}$.

For *RHC*(*n*) the number of nodes and edges are $2n^2+8n+6$ and $3n^2+10n+6$ respectively. It’s diameter is $4n+3$.

### Lemma 3

In graph $RHC(n),n>0$, the nodes $v_{11}$ and $v_{2n+2,3}$ is the only one pair of nodes at diametric distance.

### Proof

Consider the nodes $v_{11}$ and $v_{2n+2,3}$ of *RHC*(*n*). Choose an arbitrary node, say $v_{n+2,1}$ in a path joining $v_{11}$ and $v_{2n+2,3}$.

The shortest path between $v_{11}$ and $v_{n+2,1}$ is given by $v_{11} \rightarrow v_{12}\cdots \rightarrow v_{n+1,1} \rightarrow v_{n+2,1}$. The number of nodes in the path joining $v_{11}$ and $v_{n+2,1}$ is $2n+3$. This implies, $d(v_{11},v_{n+1,1} )=2n+2$.

The path between $v_{n+2,1}$ and $v_{2n+2,3}$ is given by $v_{n+2,1}\cdots \rightarrow v_{2n+2,1} \rightarrow v_{2n+2,3}$. The number of nodes in the path joining $v_{n+1,1}$ and $v_{2n+2,3}$ is $2n+1$. Clearly $d(v_{n+1,1},v_{2n+2,3} )=2n+1$.

Hence, $d(v_{11},v_{2n+2,3} )=d(v_{11},v_{n+1,1} )+d(v_{n+1,1} ,v_{2n+2,3} )=2n+2+2n+1$ $=4n+3$. In *RHC*(*n*), the nodes $v_{11}$ and $v_{2n+2,3}$ are the top and bottom node, as shown in Fig. 4. By the structure of *RHC*(*n*), for any pair of nodes $\alpha \ne v_{11}$ and $\eta \ne v_{2n+2,3}$ in *RHC*(*n*), $d(\alpha ,v_{2n+2,3} )<4n+3$, $d(v_{11},\eta )<4n+3$ and $d(\alpha ,\eta )<4n+3$. $\square$

### Theorem 3.1

$$an(RHC(n))\ge M(RHC(n))+Y$$, $$n\ge 1$$.

### Proof

Consider *RHC*(*n*). By Definition 2, the median of *RHC*(*n*) is

3 $$\begin{aligned} M(RHC(n))=16n^2+13n+5. \end{aligned}$$

First, label the center node *v* as 1. Starting from this node *v*, the remaining nodes of *RHC*(*n*) are labeled, by applying the antipodal condition. Let $\tau$ be the optimum antipodal labeling of graph *RHC*(*n*).

Then span($$\tau$$)$$=max\{\mid \tau (\alpha ) - \tau (\eta )\mid :\alpha ,\eta \in V(RHC(n)),\alpha \ne \eta \}= M(RHC(n))+Y$$.

Therefore lower bound of the antipodal number of *RHC*(*n*) is given by

$$an(RHC(n))\ge M(RHC(n))+Y$$ where $$Y=10n^3-39n^2+94n-55, n\ge 1$$. $$\square$$

### Theorem 3.2

$$an(RHC(1))=44$$.

### Proof

For *RHC*(1), $diam(RHC(1))=7$. Using (3), the median of *RHC*(1) is 34, ie., $M(RHC(1))=34$.

By Theorem 3.1, the antipodal number is

4 $$\begin{aligned} an(RHC(1))\ge 34+(10-39+94-55)=44. \end{aligned}$$

Any antipodal labeling $\tau$ of *RHC*(1) must satisfy the following antipodal labeling condition

5 $$\begin{aligned} d(\alpha ,\eta )+\mid \tau (\alpha )-\tau (\eta )\mid \ge diam(RHC(1))=7. \end{aligned}$$

For *RHC*(1), the median occurs at node $v_{24}$. Therefore, the center of *RHC*(1) is $v_{24}$. Take $\tau (v_{24} )=1$. The remaining nodes of *RHC*(1) are chosen based on their maximum distance from the already labeled node and assigned the labeling by applying the antipodal condition given by equation (5). Node $v_{43}$ is at a maximum distance from $v_{24}$. Label $v_{43}$, i.e.,$\tau (v_{43} )=4$. From $v_{43}$, node $v_{11}$ is at maximum distance. So label it, i.e., $\tau (v_{11} )=5$. Similarly, the remaining nodes of *RHC*(1) can be labeled in the following order. Starting from $v_{11}$, node $v_{31}$ is at maximum distance, hence label it as 8. From $v_{31}$, label the node $v_{25}$ as 10, and from $v_{25}$ the nodes $v_{12},v_{35},v_{13},v_{34},v_{41},v_{21}$ and $v_{42}$ are taken in this order and labeled. From $v_{42}$, label the node $v_{23}$, and from $v_{23}$ label $v_{33}$, i.e., $\tau (v_{23} )=37$ and $\tau (v_{33} )=39$. From $v_{33}$, label the remaining node $v_{32}$ as 44, which is the span of *RHC*(1). Hence $an(RHC(1))=44$. $\square$

### Theorem 3.3

For *RHC*(*n*), $$n\ge 2$$

$$\begin{aligned} an(RHC(n)) \le \left\{ \begin{array}{rcl} 8n^3+29n^2+25n+3 &{} \text{ for } &{} n=2 \\ 8n^3+30n^2+22n+5 &{} \text{ for } &{} n>2. \end{array}\right. \end{aligned}$$

### Proof

In *RHC*(*n*), the number of nodes $\mid V(RHC(n))\mid =2n^2+8n+6$ and the number of edges $\mid E(RHC(n))\mid =3n^2+10n+6$.

Let $\tau$ be an assignment of distinct non negative integers to *V*(*RHC*(*n*)) such that

6 $$\begin{aligned} \mid \tau (\alpha )-\tau (\eta )\mid =4n+3-d(\alpha ,\eta ). \end{aligned}$$

The nodes of *RHC*(*n*) are labeled as follows.

Take $\tau (v_{11} )=1$. By Lemma 3, the vertex $v_{2n+2,3}$ is diametric opposite to the vertex $v_{11}$. Therefore by antipodal condition, take $\tau (v_{2n+2,3} )=\tau (v_{11} )=1$. The remaining nodes of *RHC*(*n*) are labeled as follows.

*Case (i):* $$L_k=\{v_{kj},2\le k\le n+2\}$$.

   *Subcase (a):* If $j=1$ and $j=k+1$, then $\tau (v_{kj} )= \tau (v_{k-1,2k-1}) +4n+3-(2k-1)$.

   *Subcase (b):* If $2\le j\le 2k+1, j\ne k+1$, then $\tau (v_{kj} )= \tau (v_{k,j-1}) +4n+1$.

*Case (ii):* $$L_k=\{v_{kj},n+3\le k\le 2n+2\}$$.

By Remark 3, the number of nodes in the levels $L_{n+2}=L_{n+1},L_{n+3}=L_n\ldots L_{2n+2}=L_1$.

   *Subcase (c):* If $j=1$ and $j=k-1$, then $\tau (v_{kj} )=\tau (v_{k-1,2(k-1)+1}) +4n+3-(2k-1)$.

   *Subcase (d):* If $1\le j\le 2k+1, j\ne k-1$, then $\tau (v_{kj} )= \tau (v_{k,j-1}) +4n+1$.

Now, we claim that the above cases satisfies the antipodal condition (6).

*Case (1):* Let $\alpha ,\eta \in RHC(n)$ be the nodes in the same level and $d(\alpha ,\eta )=1$, Clearly $\tau (v)\ge diam(RHC(n))$.

From sub case(a), $\alpha =u_{k1},\eta =v_{k,k+1}$ and $\mid \tau (\alpha )-\tau (\eta )\mid =4n+3-2k+1-1$ for any *k*, $\Longrightarrow \tau (\alpha )=4n-1+\tau (\eta )\ge 4n+3$.

From sub case (b) for, $j\ne 1$, $j\ne k+1$ and $\tau (\alpha )=4n+1+\tau (\eta )-1$ $=4n+\tau (\eta )\ge 4n+3$.

The proof is similar for sub cases (c) and (d).

*Case (2):* Suppose the nodes lie in different levels and $d(\alpha ,\eta )=1$.

Let $\alpha =u_{k,k+1},\eta =v_{k+1,1},2\le k\le 2n+2$. From sub case (a), $\mid \tau (\alpha )-\tau (\eta )\mid =4n+3-2k+1-1$ $\ge 4n+3$ for any *k*.

Similarly, the antipodal condition can be verified for other sub cases.

*Case (3):* Let $2\le d(\alpha ,\eta )\le 4n+3$. Suppose $d(\alpha ,\eta )=4n+3$.

Let $\alpha =v_{11},\eta =v_{2n+2,3}$. By Lemma 3, $d(v_{11},v_{2n+2,3}) =4n+3$. Therefore $\tau (\alpha )-\tau (\eta )=0$, since the diametrically opposite nodes receive the same label.

Suppose $$d(\alpha ,\eta )\ge 2$$, $$2\le k\le 2n+2$$.

Let $$\alpha =v_{kj},\eta =v_{k,j+1}$$. Therefore $$\mid \tau (\alpha )-\tau (\eta )\mid =4n+3-d(\alpha ,\eta )>4n+3$$.

Let $$\alpha =v_{k,j},\eta =v_{k+1,j}\in RHC(n)$$, $$\mid \tau (\alpha )-\tau (\eta )\mid =4n+3-d(\alpha ,\eta )$$ $$>4n+3$$.

Hence the antipodal condition is satisfied for all the cases. In *RHC*(*n*), the node $v_{2n,2}$ receives the maximum labeling.

Therefore the upper bound of the antipodal number of *RHC*(*n*) is

$$\begin{aligned} an(RHC(n)) \le \left\{ \begin{array}{rcl} 8n^3+29n^2+25n+3 &{} \text{ for } &{} n=2 \\ 8n^3+30n^2+22n+5 &{} \text{ for } &{} n>2. \end{array}\right. \end{aligned}$$

$$\square$$

## Results and discussions

**Figure 5:**
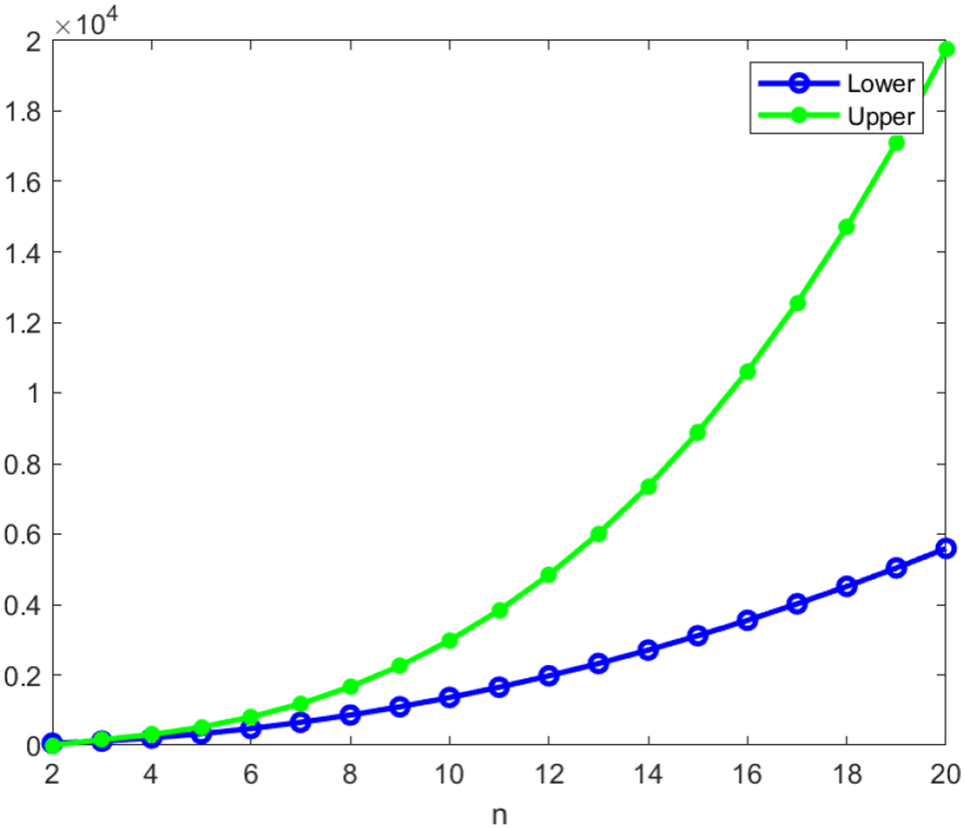
Bounds variations of *THC*(*n*).

**Figure 6:**
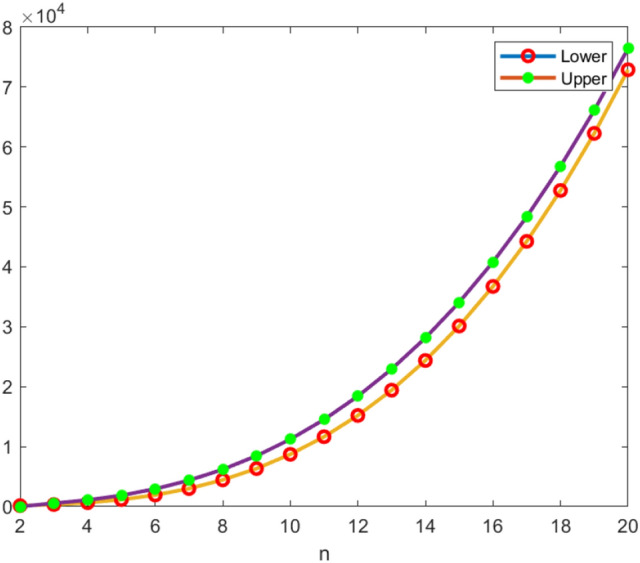
Bounds variations of *RHC*(*n*).

The bounds of *an*(*THC*(*n*)) and *an*(*RHC*(*n*)), $n\ge 2$ have been displayed in Table 1. These bounds help to determine the maximum and the minimum number of channels needed for this network to have efficient communication without interference. The graphical representations of these bounds are given in Figs. 5 and 6. These figures clearly indicate that *an*(*THC*(*n*)) and *an*(*RHC*(*n*)) increase rapidly as the dimension (*n*) of the network increases. Also, we observe that in the rhombic honeycomb network the bounds almost coincides as *n* increases, whereas the gap among the bounds of the triangular honeycomb network widens as *n* increases.

**Table 1:** Bounds of *THC*(*n*) and *RHC*(*n*).

Honeycomb networks	$THC(n), n\ge 2$	$RHC(n), n\ge 2$
Number of nodes(k)	$n^2+6n+6$	$2n^2+8n+6$
Lower bound(y)	$M(THC(n))+2n$	$M(RHC(n))+Y$
Upper bound(y)	$2n^{3}+9n^{2}+7n+5$	$8n^3+30n^2+22n+5$

## Conclusion

The channel assignment problem in wireless communication is an optimization problem in which the interference between the channels assigned have to be minimized and the service has to be provided to the maximum number of users. Many techniques are employed to solve this problem. The graph labeling method is one of the mathematical technique helps to solve the problem with low cost and time, which can be easily adopted for any adhoc networks. The honeycomb network is quite often used in communication engineering because of its structure. In our work, the derived structures from honeycomb networks are considered and bounds of *an*(*THC*(*n*)) and *an*(*RHC*(*n*)) have been determined. This bounds helps to know the optimum bandwidth needed for these networks for effective communication. Also, these bounds were plotted graphically. The problem of finding the antipodal number for different communication networks are still open for many Wireless Adhoc Networks.

## Data Availability

All data generated or analysed during this study are included in this published article.
